# Management algorithm for pulmonary vein stenosis: An evaluation of available surgical data

**DOI:** 10.1016/j.hroo.2026.03.038

**Published:** 2026-04-10

**Authors:** Johanna Bauer, Karol Chorąży, Stefan Schwarz, Shahrokh Taghavi, Clemens Aigner, Veronika Vetchy, Christian Gerges, Irene Lang, Bernhard Moser

**Affiliations:** 1 Department of Thoracic Surgery, Medical University of Vienna, Vienna, Austria; 2 Department of Biomedical Imaging and Guided Therapy, Medical University of Vienna/General Hospital Vienna, Vienna, Austria; 3 Division of Cardiology, Department of Internal Medicine II, Vienna General Hospital, Medical University of Vienna, Vienna, Austria; 4 Comprehensive Center for Chest Diseases, Medical University of Vienna, Vienna, Austria

**Keywords:** Pulmonary vein stenosis, Radiofrequency ablation, Atrial fibrillation, Surgical intervention for pulmonary vein stenosis, Major thoracic surgery, Treatment algorithm

## Abstract

**Background:**

Pulmonary vein stenosis (PVS) is a rare complication after radiofrequency ablation for patients with atrial fibrillation. However, the impact of a high-grade stenosis on a patient’s hemodynamic situation and quality of life can be severe. Percutaneous balloon angioplasty or stent implantation are interventional treatment options for severe and symptomatic PVS. Owing to significant postinterventional restenosis rates, the management of PVS remains challenging, and the investigation of other options, such as surgical correction, is warranted.

**Objectives:**

In this study, a structured literature review on surgical treatment strategies for PVS was performed. Based on these findings and multidisciplinary clinical experience, we developed a pragmatic management algorithm to guide individualized treatment decisions for patients with severe or recurrent PVS.

**Methods:**

A literature review was conducted using PubMed, following the population, intervention, comparison, and outcome framework. A treatment algorithm was designed as a decision flowchart.

**Results:**

The literature search identified 7 publications on cardiothoracic surgical techniques for the treatment of PVS, describing 11 cases. Although the most common surgical approach was pericardial patchplasty, sutureless techniques and a combination of operations, including endarterectomy, bypass, widening plasty after stent implantation, suturing the vein directly to the left auricle, or the use of pulmonary homograft tissue, were also described in the literature.

**Conclusion:**

Managing PVS is challenging, especially in cases of restenosis. These patients should be discussed in a multidisciplinary setting, including cardiology, radiology, and thoracic surgery. We propose a treatment algorithm that may aid in individual decision making and serve as a starting point for future discussions.

## Graphical abstract

**Figure:**
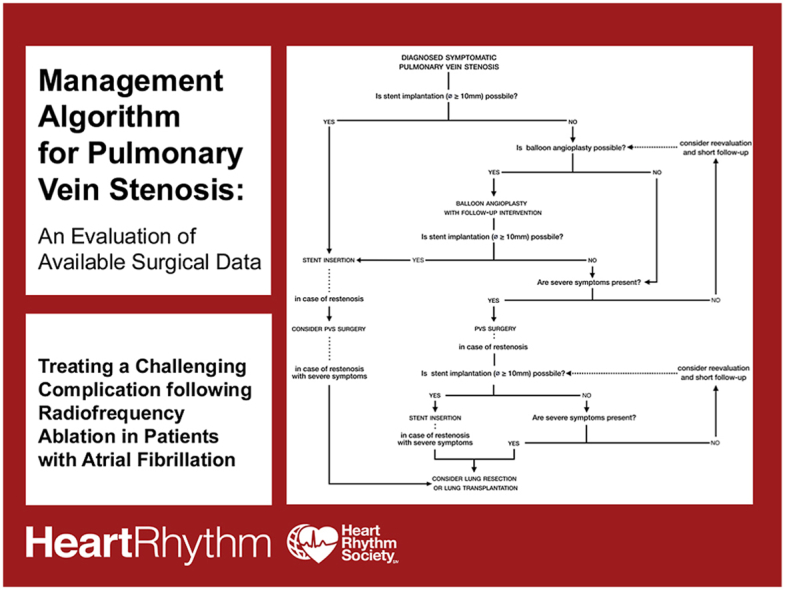

**Key Findings**

▪ If adequate stent implantation fails or restenosis occurs and the patient experiences severe symptoms, pulmonary vein stenosis surgery should be considered.
▪ Setting the indication for surgery on a case-by-case basis requires interdisciplinary assessment with expertise from cardiology, radiology, and cardiothoracic surgery.
▪ Surgeries such as pulmonary vein patch angioplasty should be reserved for centers with experience in major thoracic surgery under cardiopulmonary bypass.
▪ Our proposed treatment algorithm may assist in individual treatment decisions and serve as a foundation for future treatment discussions.

## Introduction

Radiofrequency ablation (RFA) is an established treatment option for atrial fibrillation. Pulmonary vein stenosis (PVS) is a rare complication (0.5%–4%) after RFA and results from myocardial fibrosis, vascular wall proliferation, and thrombus formation.^1^

The severity depends on the degree of narrowing of 1 or more pulmonary veins (PVs) and can range from mild (<50%) and moderate (50%–70%) to severe (>70%).^2^

Although 30%–40% of patients may show mild stenosis on the postinterventional computed tomography, most remain asymptomatic. However, the impact of a severe stenosis on a patient’s hemodynamics and their quality of life can be profound. Owing to the stenosis, the patient may develop pulmonary hypertension or infarction, dyspnea, reduced exercise capacity, hemoptysis, chronic cough, and chest pain. Given that the clinical presentation of severe PVS is often nonspecific, up to 35% of patients are initially misdiagnosed.^1^ A diagnostic delay can lead to PV occlusion at the left atrial connection. However, noninvasive imaging, such as a computed tomography scan alone, may be insufficient to diagnose PV occlusion accurately. Using invasive transfemoral catheterization, Fender et al^5^ identified residual microchannels in 34% of patients initially diagnosed as having PV occlusion.^1^, ^2^, ^3^, ^4^, ^5^

Percutaneous balloon angioplasty (BA) and stent implantation are common treatments for severe and symptomatic PVS. Although these percutaneous interventions are widely available, data on surgical options for PVS are scarce. The surgical strategy includes patch angioplasty, bypass, endarterectomy, and sutureless techniques, which are more commonly used in pediatric PV reconstruction.^6^

## Materials and methods

A selective literature review was conducted to identify publications on the cardiothoracic surgical management of PVS stenosis using PubMed and a combination of keywords such as stenosis, pulmonary vein, pulmonary vein stenosis, adults, surgical repair, surgery, stenosis, pulmonary vein (Medical Subject Headings terms), and multimodal. In addition, filters on age (“adults: +19”) and publication date (2005–2025) were used. Papers not written in English and publications on pediatric PVS reconstructions were excluded. The analysis was performed following the population, intervention, comparison, and outcome framework^7^:

• Population: adult patients with PVS after ablation for atrial fibrillation
• Intervention: surgical treatment options
• Comparison: percutaneous interventions such as BA or stent implantation
• Outcome: restenosis or reintervention rate
The research question was as follows: what is the outcome of surgical treatment options in adult patients with PVS after ablation for atrial fibrillation?

There were no limitations on the type of research that could be included in the study. However, owing to the rarity of the topic, only case reports were available.

The number of patients, age, number of veins, previous interventions for PVS, type of surgery, incision, surgical complications, follow-up, restenosis rate, reintervention rate, and type of reinvention were assessed for each study. Surgical outcome was analyzed by the restenosis or percutaneous reintervention rate. No additional data were obtained from the individual study investigators. Possible biases in the studies include missing data owing to short follow-up, selection bias, and reporting bias.

The proposed treatment algorithm is based on our institutional experience and publications on this topic, including those by Almakadma et al,^1^ Hill et al,^8^ Schoene et al,^9^ Van Putte et al,^10^ Suntharos et al,^11^ and Fender et al.^5^^,^^12^

The algorithm is structured as a decision flowchart that begins at the top. Although it reflects the available scientific evidence, its limitations must be acknowledged, including the absence of prospective data and randomized studies.

Ethics approval was not required for this study, as confirmed by a formal waiver issued by the chair of the Medical University of Vienna’s ethics committee.

## Results

**Figure 1:**
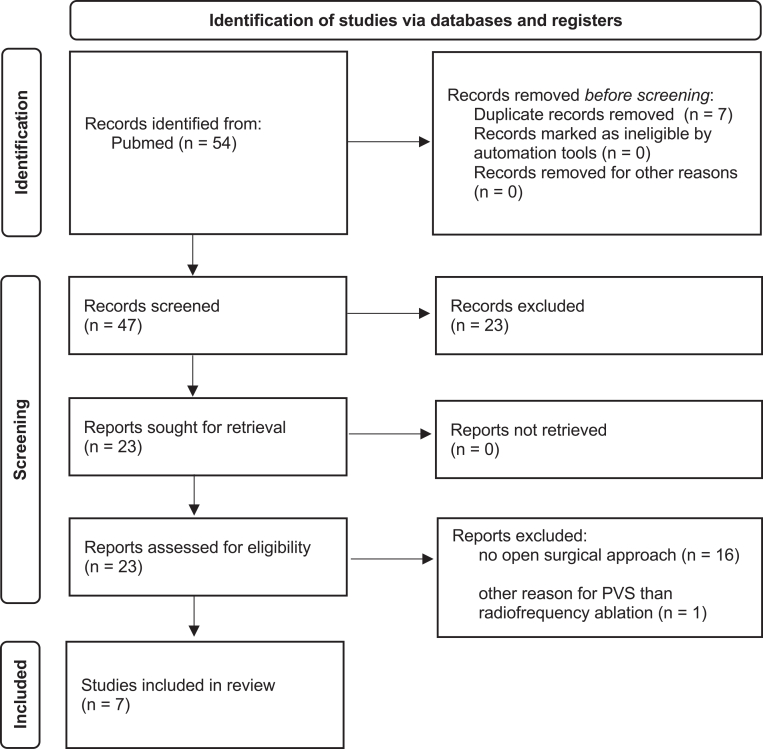
Preferred Reporting Items for Systematic Reviews and Meta-Analyses flow diagram. Of the 54 identified studies, 7 were included in the review. Adapted from Page et al.^13^ PVS = pulmonary vein stenosis.

In our literature search, we identified 54 relevant publications. 7 publications were ultimately included in the final review (Figure 1).^13^ 24 were excluded because they did not address PVS in adults, and 1 was excluded because PVS was not based on RFA. 16 publications described topics such as PVS assessment, diagnostics, imaging, and percutaneous interventions, but were excluded for not describing open surgery. Although some of the most prominent publications on percutaneous treatments include up to 172 patients, data on surgical approaches are scarce, consisting only of case reports.

The 7 publications describe different techniques for achieving adequate venous drainage (Tables 1 and 2). Hara et al^14^ and Schoene et al^9^ performed the operation using pericardial patchplasty. Tarui et al^6^ and Hirota et al^16^ described sutureless techniques by which the pericardium is placed over the opened stenotic veins without direct closure of the venous incision line. The latter presented a case with a complex redo surgery: Hirota et al^16^ operated on a patient who had previously undergone pericardial patchplasty after developing restenosis after previous stent implantation. No postoperative complications were mentioned in the case report. Van Putte et al^10^ performed a widening plasty in a patient who had previously undergone stent implantation for PVS. Shimojima et al^15^ and Patel et al^17^ each described 1 case but used a combination of techniques, including bypass, endarterectomy, patchplasty, suturing the vein directly to the left auricle, and the use of pulmonary homograft tissue.

**Table 1:** Summary of current available data on thoracic surgery in PVS surgery type and patient specifications

Thoracic surgery in PVS							
Author	Surgical technique	Age	Total numberof patients	Total number ofstenotic veins	Total number ofoccluded veins	Previous interventions for PVS	Incision
Hara et al^14^	Pericardial patchplasty	73	1	2	2	0	Re-Sternotomy
Schoene et al^9^	Pericardial patchplasty	49 ± 6	5	13	0	2 PV restenosis after BA, 2 failed angioplasties, 1 PV restenosis after urgent patchplasty for BA-induced rupture	Sternotomy
Shimojima et al^15^	Surgical bypass operation, endarterectomy, pericardial patchplasty	68	1	2	2	0	NA
Tarui et al^6^	Sutureless technique	37	1	4	0	0	NA
Van Putte et al^10^	Widening plasty after stent implantation	55	1	2	0	3 BA and 1 stent implantation LSPV, 1 stent implantation LIPV	Thoracotomy
Hirota et al^16^	Sutureless technique for restenosis after pericardial patchplasty	41	1	3	0	Stent implantation LSPV, then restenosis and pericardial patchplasty (followed by restenosis after 5 mo)	NA
Patel et al^17^	LIPV was sutured to the left auricle; RSPV was bridged to the RMLV using pulmonary homograft tissue.	21	1	1	2	0	NA

BA = balloon angioplasty; LIPV = left inferior pulmonary vein; LSPV = left superior pulmonary vein; NA = not available; PV = pulmonary vein; PVS = pulmonary vein stenosis; RMLV = right middle lobe vein; RSPV = right superior pulmonary vein.

**Table 2:** Summary of current available data on thoracic surgery in PVS complications and restenosis and reintervention rates

Thoracic surgery in PVS						
Author	Surgical technique	Surgical complications	Follow-up,mo	Restenosisrate	Reinterventionrate	Type ofreintervention
Hara et al^14^	Pericardial patchplasty	Revision surgery owing to bleeding	NA	NA∗	NA	NA
Schoene et al^9^	Pericardial patchplasty	1 revision for pericardial effusion	60 ± 69†	38%	7.7%	BA
Shimojima et al^15^	Surgical bypass operation, endarterectomy, pericardial patchplasty	NA	2	NA‡	NA	NA
Tarui et al^6^	Sutureless technique	NA	6	0%	0	NA
Van Putte et al^10^	Widening plasty after stent implantation	NA	3	0%§	NA	NA
Hirota et al^16^	Sutureless technique for restenosis after pericardial patchplasty	NA	7	NA	NA	NA
Patel et al^17^	LIPV sutured to the left auricle, RSPV was bridged to RMLV using pulmonary homograft tissue	NA	18	0%	NA	NA

LIPV = left inferior pulmonary vein; LSPV = left superior pulmonary vein; NA = not available; PV = pulmonary vein; PVS = pulmonary vein stenosis; RMLV = right middle lobe vein; RSPV = right superior pulmonary vein.

∗ Hara et al^14^ described a subclinical stenosis of 1 pulmonary vein without further specifications.

† Mean of a total of 5 patients (the individual values are mentioned in the paper).

‡ Shimojima et al^15^ reported that a transthoracic echocardiography after 2 months indicates the development of restenosis in 1 vein and 1 bypass.

§ Van Putte et al^10^ reported that the computed tomography scan 3 months postoperatively showed a fully patent LSPV and a slightly tapered LIPV without further specification.

The largest case series by Schoene et al^9^ included 5 patients, all of whom underwent pericardial patchplasty via sternotomy between 2004 and 2016 for a total of 13 stenotic veins. 38% of these veins developed restenosis, and 1 vein (7.7%) required reintervention via BA owing to restenosis. Schoene et al^9^ (60 ± 69 [n = 5]) and Patel et al^17^ (18 months) had the longest reported follow-up period. In 4 papers, the reported follow-up time after surgery was short (≤7 months),^6^^,^^10^^,^^15^^,^^16^ and 1 paper did not specify a follow-up period.^14^ Combining the follow-up periods of these 10 documented patients, the mean follow-up period is 33.5 months, whereas the median is 6.5 months (ranging from 2 to 149 months).

According to the literature, stent implantation has the lowest restenosis rates compared with BA and thoracic surgery. Based on the limited data available, there is no published peer-reviewed evidence on a surgical approach to PVS after RFA.^18^

## Discussion

Owing to the rarity and variety of these surgical approaches and based on the limited data available, there is no high-level evidence for recommending open surgery.^18^ Setting the indication for surgery on a case-by-case basis requires interdisciplinary assessment with expertise from cardiology, radiology, and cardiothoracic surgery.

Schoene et al^9^ published the largest case series yet with 5 patients who underwent patchplasty via sternotomy with either bovine pericardium or polytetrafluoroethylene (Gore-Tex®). 3 of 5 patients in the study, or 5 of the 13 operated PVs (38%), developed restenosis within 15 months after the surgery. However, the median follow-up of the 7 publications identified through our literature review was only 6.5 months. This duration might be too short to accurately assess long-term outcomes, such as restenosis rates.

What if surgery results in restenosis? Hill et al^8^ reported about a patient who underwent surgical reconstruction with a pericardial patch and postoperatively developed occlusion. Although recanalization and stenting (6 mm) were initially successful, the stented PV also became chronically occluded, making further recanalization not possible anymore.

In 2023, our department performed pericardial patchplasty via clamshell incision under cardiopulmonary bypass in a patient with severe PVS on both sides. The patient developed restenosis 12 months after initial surgery and received percutaneous stent implantation in the right and left superior PVs, without any adverse events (Table 3).^12^

**Table 3:** Complicated case in the Medical University of Vienna complications and restenosis and reintervention rates

Thoracic surgery in PV stenosis						
Hospital	Surgical technique	Surgical complications	Follow-up, mo	Restenosis rate	Reintervention rate	Type of reintervention
Medical University of Vienna	Pericardial patchplasty	NA	26	75%	50%	Stent implantation RSPV and LIPV

12months after initial surgery.

LIPV = left inferior pulmonary vein; NA = not available; PV = pulmonary vein; RSPV = right superior pulmonary vein.

Given the limited data on PVS surgery, the question remains in which cases surgery should be considered and whether upfront surgery can be seen as a valid first choice without previous intervention or as a last resort. Current data suggest that the lowest restenosis rate (20%) occurs after stent implantation, which should therefore be considered as first-line treatment for PVS.^1^ Fender et al^5^ compared BA and stent implantation in 124 patients and found that BA results in a higher restenosis rate than stent implantation (57%–27%) with a median time to restenosis of 0.3–0.8 years. Major adverse events such as PV perforation or cardiac tamponade occurred in 3.5% of patients. A meta-analysis by Almakadma et al^1^ on 4 studies comparing restenosis after stenting with BA showed comparable results with restenosis rates of 54% (BA) to 20% (stent). The pathophysiology of restenosis in BA is suspected to be based on elastic recoil, whereas the cause of restenosis after stenting may be intima hyperplasia.^5^

However, choosing an appropriate stent size remains challenging given the highly compliant surrounding tissue and the diameter of stenosis. Stent length requires careful consideration to avoid possible overhang into the left atrium and the obstruction of segmental vein branches.^5^ Suntharos et al^11^ compared different stent sizes and found that larger diameters play a crucial role in reducing restenosis rates. Freedom from reintervention at 5 years was 9% for the <7 mm stent group, 79% for the ≥7 mm stent group, and even 83% for the ≥10 mm stent group. If the diameter of the stenosis does not allow for the implantation of a larger stent, preceding BA should be considered.^8^ Drug-eluting stents for PVS with comparable diameters are currently not available but may further reduce the restenosis rate in the future.

Nevertheless, approximately 20% of patients develop restenosis after stent implantation. If restenosis does occur with a high burden of symptoms, which treatment options can still be considered for the patient? Van Putte et al^10^ published a case report about a 55-year-old female patient who received stent implantation in the left superior and inferior PV for PVS after catheter ablation. 1 week after the stenting, she developed symptomatic occlusion, and the indication was set to perform a bilateral video-assisted thoracoscopic maze operation, followed by a 15 cm left-sided thoracotomy. Under cardioplegia, the left superior and inferior PVs were opened, and the stents showed a complete re-endothelialization. The stents were opened longitudinally, and a widening plasty was performed with a continuous suture along the stent. 3 months of follow-up showed both veins to be patent, but long-term data are not available in this case.^10^ Hirota et al^16^ also described a complex redo surgery in a patient who initially underwent stent implantation and received pericardial patchplasty. After 5 months, the patient developed restenosis again and was revised using a sutureless technique. Schoene et al^9^ described in their case series that they did not perform surgery on 1 of the PVS owing to preceding advanced stent implantation.

Current literature suggests that PVS may progress to occlusion if left untreated.^4^ However, there are limited or no data on the efficacy of noninvasive therapy with anticoagulation and on the consideration of only symptomatic treatment until collateralization potentially restores sufficient venous outflow.^19^

Several limitations of this study should be acknowledged. There are no randomized studies on the management of PVS, comparing stent implantation, BA, and surgery available today. Given the small number of case reports on surgical management and the retrospective nature of this study, the analysis and treatment algorithm are biased by the level of evidence. Surgical cases with unfavorable outcomes may also be underrepresented in the literature. However, given the limited data available, the treatment of PVS poses a challenge for clinicians, supporting the need for a structured overview of the existing knowledge and the development of a treatment algorithm.

### Treatment algorithm

**Figure 2:**
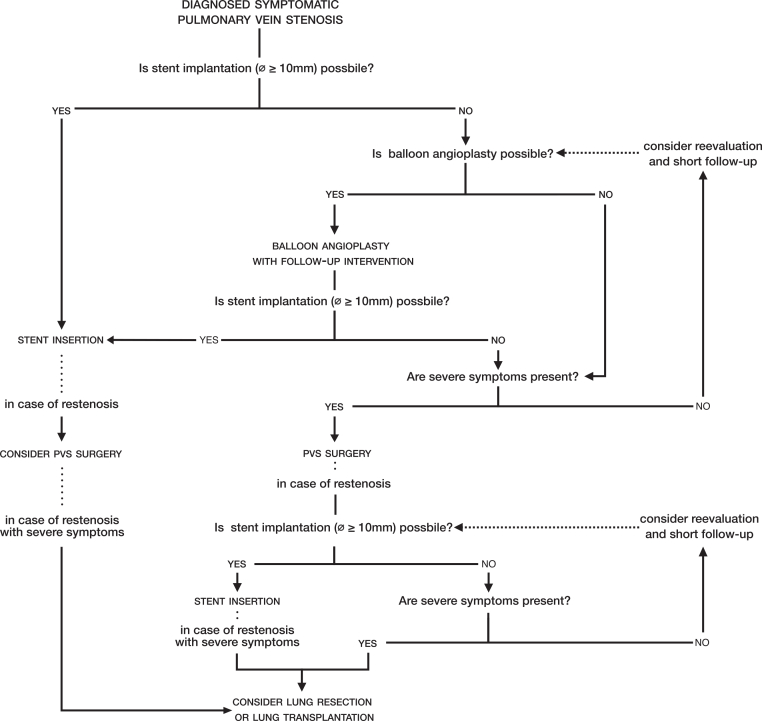
Proposed guide for interdisciplinary decision making. The flowchart is designed as a top-to-bottom decision tree that starts with a diagnosed and symptomatic pulmonary vein stenosis. Each stage corresponds to a specific clinical question, such as whether stent insertion with a diameter of >10 mm is possible. The *arrows* recommend a treatment approach, and the *dotted line* indicates further options in the case of restenosis. PVS = pulmonary vein stenosis.

Our treatment algorithm for managing PVS was developed using available clinical evidence and expert physician experience (Figure 2). In collaboration with the department of cardiology, the department of thoracic surgery regularly holds interdisciplinary boards and the institution acts as a referral center in the treatment of pulmonary hypertension including pulmonary endarterectomy. Any PVS cases are also presented in this board.

The algorithm is structured as a decision flowchart, beginning at the top. Although it reflects the available scientific evidence, its limitations must be acknowledged, including the absence of prospective data and randomized studies. Our proposed algorithm was designed to provide clinicians with a structured approach for the treatment of PVS and may guide individual decisions regarding this rare yet very complex pathology. It combines our institutional clinical experience with evidence from several publications, including Schoene et al,^9^ Hill et al,^8^ Suntharos et al,^11^ Van Putte et al,^10^ and Fender et al,^5^ which have outlined important factors for treatment decision making.

Every patient should be discussed in a multidisciplinary board, and clinicians should consider patient characteristics such as PV occlusion, age, and multimorbidity. The algorithm starts with a high-grade, symptomatic PVS. Given its current restenosis rate, stent implantation should be considered as the first-line treatment option.^1^ A key factor here is the size of the implanted stent, given that a diameter of >10 mm leads to significantly more freedom from reintervention (>5 years: ≥10 mm 83%; ≥7 mm for 79%; <7 mm for 9%). If a stent that size cannot be implanted, primary surgical correction or BA should be considered instead of using a stent smaller than 10 mm in diameter.^8^^,^^11^

If adequate stent implantation fails or restenosis occurs and the patient complains about severe symptoms such as dyspnea, decreased physical performance, or chest pain, surgery might be considered. Van Putte et al^10^ and Hirota et al^16^ demonstrated the possibility of surgery even after stenting.

If restenosis occurs after an open surgical approach, the patient should be evaluated for percutaneous stenting.^8^ If surgery after stent insertion, or vice versa, fails to achieve patent PVs, lobectomy may be considered in cases where secondary symptoms such as hemoptysis persist. However, an anatomic lung resection will only be feasible in a minority of cases of PV restenosis affecting only 1 vein and after careful evaluation of potential pulmonary hypertension and its associated perioperative risks.^5^^,^^8^^,^^20^, ^21^, ^22^

If the stenosis extends into and beyond the segmental PVs, neither stenting nor surgery may restore adequate blood flow to the left atrium. In the case of a bilateral stenosis and a significantly reduced functional status, lung transplantation might present as an ultima ratio.^2^ Despite our department being 1 of the largest lung transplantation centers in Europe, we have no documented case of lung transplantation for PVS resulting from RFA. Nevertheless, transplantation remains a conceivable option of last resort, particularly in cases where progressive scarring involves segmental and subsegmental veins that are inaccessible to both interventional and surgical therapies.

**Figure 3:**
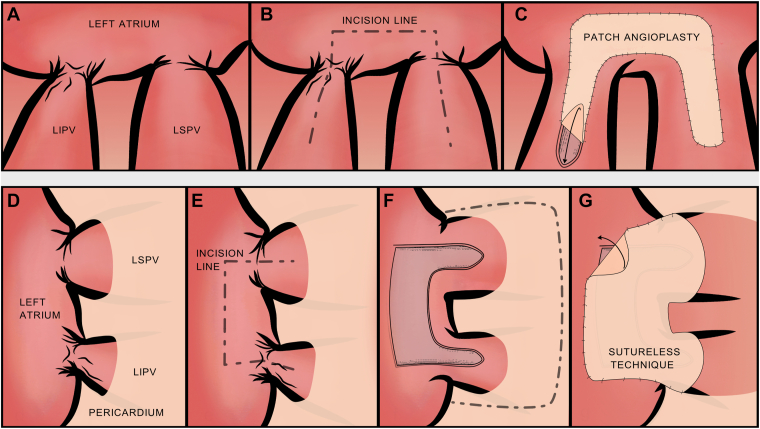
Illustration of different surgical techniques. **A–C:** Clamshell view of the left atrium showing the stenosis, incision line, and the inserted patch. **D–G:** Sternotomy view of the left atrium showing the stenosis, the incision line for the left atrium, and a sutureless technique (original illustration by the author). LIPV = left inferior pulmonary vein; LSPV = left superior pulmonary vein.

Owing to limited data on surgical management and the heterogeneity of the existing case reports, our treatment algorithm does not recommend a specific surgical approach. Although pericardial patchplasty is the most common procedure in the current literature, other methods, including sutureless techniques, can be considered (Figure 3).^9^^,^^14^^,^^15^ The specific approach may also depend on the surgeon’s experience in major cardiothoracic surgery under cardiopulmonary bypass.

## Conclusion

In an era of widespread availability for percutaneous cardiac interventions, a small percentage of patients develop a rare but demanding complication, presenting the cardiothoracic field with the challenge of finding surgical therapy options with only a small pool of cases to guide them. Although BA or stent insertion can also have major adverse events, the possible complications and the extent of open surgery should be carefully considered. The indication for surgical intervention should be discussed in an interdisciplinary board, together with thoracic surgery, cardiology, and radiology. Surgeries such as PV patch angioplasty should be reserved for centers with experience in major thoracic surgery under cardiopulmonary bypass.

Ultimately, larger datasets and further research are required to set proper guidelines for the management of PVS. Our proposed treatment algorithm may assist in individual treatment decisions and serve as a foundation for future treatment discussions.

## Disclosures

The authors have no conflicts of interest to disclose.
